# Exogenous ketone bodies and the ketogenic diet as a treatment option for neurodevelopmental disorders

**DOI:** 10.3389/fnut.2024.1485280

**Published:** 2024-12-19

**Authors:** Naomi Elyse Omori, Mantas Kazimieras Malys, Geoffrey Woo, Latt Mansor

**Affiliations:** ^1^Health Via Modern Nutrition Inc. (H.V.M.N.), San Francisco, CA, United States; ^2^Department of Psychological Medicine, King’s College London, Institute of Psychiatry, Psychology & Neuroscience, London, United Kingdom

**Keywords:** autism, ASD, ADHD, neurodevelopment, ketones, ketone bodies, ketogenic diet, BHB

## Abstract

**Background:**

Despite being the most prevalent neurodevelopmental disorders, there are comparatively few treatment options available to patients presenting with autism spectrum disorder (ASD) and attention deficit hyperactivity disorder (ADHD). The ketogenic diet has historically shown therapeutic utility in treating refractory epilepsy, an adjacent neuropsychiatric condition, in children, adolescents and adults. The following review explores preclinical and clinical literature focusing on the therapeutic potential of the ketogenic diet and exogenous ketone body supplementation in treating common neurodevelopmental disorders.

**Method:**

A narrative review of extant literature was conducted across the domains of perinatal nutrition, ASD, and ADHD. Preclinical and clinical studies focusing on the effect of either the ketogenic diet or exogenous ketone supplementation as a treatment option were included for review.

**Results:**

14 preclinical and 10 clinical studies were included for discussion. Data supporting the use of a ketogenic intervention for neurodevelopmental disorders is mixed. High heterogeneity in study design was noted for preclinical models, ketogenic intervention, and outcomes measured.

**Conclusion:**

Studies evaluating ketogenic interventions for neurodevelopmental disorders remain in their infancy in terms of both the depth and scope of available literature. The safety and tolerability of ketogenic diets and supplements means there would be value in exploring their effectiveness further in clinical studies.

## Introduction

Since the first clinical usage of the ketogenic diet to treat refractory epilepsy almost a century ago (1), a slowly proliferating body of evidence has emerged demonstrating the diet’s therapeutic potential in a range of adjacent neuropsychiatric disorders including depression, anxiety, schizophrenia (2), neurodegenerative diseases such as Alzheimer’s (3), and complications arising from traumatic brain injuries (4). The following narrative review explores the current evidence for and against the use of the ketogenic diet and exogenous ketone supplementation in treating neurodevelopmental conditions, including autism spectrum disorder (ASD) and attention deficit hyperactivity disorder (ADHD). The impact of these interventions during the perinatal period on neurodevelopment are also reviewed.

Treatments for neurodevelopmental disorders are unique among neuropsychiatric disorders in that they are often initiated in childhood. Paediatric populations frequently inspire a tendency for clinicians to be more considerate of holistic well-being, which is often reflected in a greater sensitivity towards short and long-term side-effects; short-term side effects can certainly limit compliance, while undesirable adverse effects long-term may deleteriously impact quality of life. Novel therapeutic agents for neurodevelopmental disorders, therefore, are of particular interest to patients and clinicians considering the highly limited scope of treatments currently available. ADHD is dominantly managed by central nervous system stimulants such as methylphenidate, although they can be contraindicated in the presence of certain health problems (e.g., heart disease) and can cause further physical and neuropsychiatric side-effects. However, there are no standard treatments for ASD, and symptoms are generally managed with supportive therapies like behavioural therapy (CBT), psychotherapy, or parenting skills training. In recent years, an increased understanding of the potential aetiological mechanisms of neurodevelopmental disorders has failed to translate into the development of any viable novel treatment modalities. Ketogenic protocol, which feature good safety and tolerability, thus emerge as a potential avenue of treatment.

The benefit of the ketogenic diet lies in its ability to stimulate a state of ketosis, a term that broadly refers to metabolic states where serum levels of ketone bodies are elevated. Ketone bodies (KBs), including *β*-hydroxybutyric acid (BHB) and acetoacetate (AcAc), are naturally occurring carbonyl-containing compounds produced in the liver from fatty acids via ketogenesis. Elevating the circulatory concentration of ketone bodies enables more ready access to an alternative metabolic substrate to the dominant compound glucose. Normal circulating levels of serum KBs in fed adults on a normal diet are typically less than 0.5 mM/L. Ketosis is thus broadly defined as where serum levels of KBs are elevated above 0.5 mM/L. Two types of ketotic states are differentiated, one being non-pathological physiological ketosis and the other being pathological ketoacidosis. In physiological ketosis, the rate of ketone production and consumption are balanced. Clinical guidelines usually define physiological ketosis as serum KB levels of 1–3 mM/L. By contrast, ketone production and consumption rates in ketoacidosis are disbalanced leading to unsustainably high levels of KBs, which in turn cause blood acidosis (i.e., serum HCO_3_^−^ ≤ 20 mM/L and/or venous pH ≤ 7.35). Pathological ketoacidosis generally occurs secondary to existing pathologies such as diabetes or chronic alcohol abuse, or as an adverse effect of certain active pharmaceutical agents (e.g., SGLT2 inhibitors, salicylates) and toxins (e.g., methanol, acetone). Clinical guidelines generally consider serum KB levels greater than 3 mM/L as being indicative of ketoacidosis; however, it should be noted that experiments focusing on the therapeutic value of physiological ketosis often report KB levels that impinge upon clinical guidelines for ketoacidosis, with some studies in fasted humans reporting stable levels ranging from 4 to 9 mM/L (5–7). It is thus reiterated that physiological ketosis refers to a biochemically stable elevation of circulating KBs.

Physiological ketosis may be achieved by either fasting, dietary modification, or exogenous supplementation. In adults and older children, fasting can induce ketosis in 12–18 h, although this method is not always feasible where there is a desire to maintain ketosis long-term. The most well-established pattern of dietary modification, which has been used to treat refractory epilepsy for almost a century, is a high-fat, low-carbohydrate diet known as ‘the ketogenic diet’ (KD). It utilises a 4:1 ratio of fat:(protein + carbohydrates) or approximately a 17:3:1 ratio of fat:protein:carbohydrate. A less restrictive alternative developed in the early 2000s is the modified Atkins diet (mAD) (8), which enables greater carbohydrate intake in a 1.5:1 ratio of fat:(protein + carbohydrates) or 6:3:1 ratio of fat:protein:carbohydrate (9).

Exogenous ketones can also be used to supplement (or potentially instead of) dietary modification patterns. Medium chain triglycerides (MCTs) are a class of KB precursors comprising triglycerides with aliphatic tails featuring 6–12 carbon atoms that can be extracted from readily available sources like palm kernel or coconut oil. They were first used as part of a ketotic regime by Huttenlocher et al. (10) where 60% of caloric intake was derived from MCTs and the remaining calories were distributed across a 1:1:2 ratio of fat:protein:carbohydrate. Today, variants of the KD, mAD, and MCT supplemented diets are still used in clinical practice under the guidance of dietitians.

More recently, novel ketone ester formulations have been able to reliably induce physiological ketosis without the need for further dietary modification. Some examples of ketone esters and derivatives include the commercialised (*R*)-3-Hydroxybutyl (*R*)-3-hydroxybutyrate ketone monoester (11) and bis-hexanoyl-(R)-1,3-butanediol, and chirally pure R-1,3-butanediol standalone (12–15). Studies have demonstrated that 1,3-butanediol ketone esters can reliably produce dose-dependent ketosis of 1–7 mM with minimal adverse side effects (15). Despite showing good safety and tolerability, exogenous ketone ester formulations have yet to gain widespread traction in clinical trials studying the therapeutic benefits of ketosis.

In rats fed on a non-KD chow of 4.2:1.7:1 carbohydrate:protein:fat, MCT induces physiological ketosis within 60 min that is sustained for 8 h while synthetic ketone ester preparations [*R,S*-1,3-butanediol acetoacetate diester (BD-AcAc_2_)] induce physiological ketosis within 30 min that is sustained for 8 h (16). Rapid induction of ketosis within 30 min has also been demonstrated for ketone salts (4.2% concentration βHB mineral salts) with sustained elevation following continuous feeding of the ketone salt solution for 4-weeks (17).

## Perinatal nutrition

Perinatal stress and metabolic dysfunction are known to be associated with an increased prevalence of neurodevelopmental differences (18). Prenatal adverse events or environments including maternal stress, toxicological exposure, malnutrition, and infection have all been associated with increased rates of schizophrenia, depression, ASD and ADHD in offspring (19, 20). As such, there is some interest in understanding whether maternal interventions can be employed to reduce the risk of neurodevelopmental conditions in offspring.

Early metabolomic studies indicate that neurodevelopmental conditions may be detectable as early as gestation. A retrospective study of serum from mothers with children later receiving a diagnosis of ASD taken during pregnancy was able to identify characteristic metabolite concentrations in numerous metabolic pathways including glycosphingolipid biosynthesis and metabolism, N-glycan and pyrimidine metabolism, bile acid pathways, and C21-steroid hormone biosynthesis and metabolism, which have been implicated in studies of ASD (21). Similarly, a study by Courraud et al. (22) claimed to observe characteristic metabolic profiles from neonate biochemical samples that were later diagnosed with ASD. Such early data would imply that at least some cases of neurodevelopmental deficit are present from the early days of inception, presenting a potential opportunity for remediation *in utero* via changes to maternal nutrition or potentially pharmacological intervention.

The neuroprotective effects of the ketogenic diet in adults and children have been reported for refractory psychiatric disease, neurodegenerative disease, and in the following sections of this review, early childhood neurodevelopmental disease. A natural assumption might follow that administration of the ketogenic diet in prepartum mothers might also yield some neuroprotective effect. Instead, current clinical guidance tends to perceive ketogenic diets in pregnancy as risky and rather recommends avoidance of diets promoting elevation of ketone bodies, particularly in women with gestational diabetes. These recommendations stem in part from studies of maternal diabetes and pathological diabetic ketoacidosis, which are frequently associated with poorer outcomes (23). Less clearly defined, however, is the impact of non-pathological maternal physiological ketosis on offspring.

Sussman et al. (24–26) conducted a series of studies on pregnant CD-1 mice fed a 4:1 ketogenic diet (67.4% fat, 0.6% carbohydrate, and 15.3% protein wt/wt) 30 days prior to mating. If pregnancies were brought to term, pups exposed to ketosis *in utero* were fostered by dams fed a standard diet rather than the original mothers due to complications of ketoacidosis. Embryos or pups were then subjected to various forms of testing. In all cases, alterations to neurodevelopment were observed. In one study, embryos exposed to a ketogenic diet *in utero* were imaged with optical projection tomography at day 13.5 of gestation (E13.5) and with magnetic resonance imaging (MRI) at day 17.5 (E17.5) of gestation (24). On average, embryos exposed to a ketogenic diet were volumetrically larger at E13.5 with a larger heart but smaller brain, pharynx, cervical spinal cord, hypothalamus, midbrain, and pons than the group exposed to a standard control diet. At E17.5, ketogenic diet-exposed embryos were on average smaller volumetrically, with a smaller heart and thymus but larger cervical spine, thalamus, midbrain and pons. In a cerebral imaging of pups sacrificed at either 11.5 or 21.5 days postnatal, anatomical differences were also seen (25). By 21.5 days, retarded growth was observed compared to controls including a relative bilateral decrease in the cortex, fimbria, hippocampus, corpus callosum and lateral ventricle, but a relative volumetric enlargement of the hypothalamus and medulla. A later study using the same diet protocol subjected CD-1 pups exposed to a ketogenic diet to behavioural testing as well as magnetic resonance imaging of the brain (26). Pups exposed to the ketogenic diet were found to perform worse in open-field, forced-swim, and exercise wheel testing. While the whole brain volume wasn’t found to be significantly different between the ketogenic-exposed group and control group, the ketogenic group was found to have a bilateral increase in the relative volume of the frontal cortex, cerebellum, and primary somatosensory cortex, and a bilateral decrease in relative volume in the hippocampus, striatum, motor cortex, and auditory cortex.

Rugiel et al. (27) performed an FTIR microspectroscopy study on brain slices of Wistar pups exposed to a ketogenic diet *in utero* and compared them to brain slices from pups whose mothers were fed a normal diet. Providing some structural insight, the study’s main strength was the spatially localised quantitative analysis of biological macromolecules including protein, lipids, cholesterol, and compounds containing phosphate and carbonyl. Pups were sacrificed at 2, 6, and 14 days to provide some longitudinal insight in early neonatal brain development. At birth, pups from dams fed a ketogenic diet had a lower body mass although this was restored with feeding a normal diet by day 14 postnatal. At days 2 and 6 no major biochemical differences were seen in the pups exposed to a ketogenic diet *in utero*, with the exception of a relatively higher proportion of phosphate-containing compounds in the cortical white matter of 2 day old pups. At day 14 more pronounced discrepancies are seen in the ketosis exposed pups including increased levels of carbonyl groups in cortex, decreased lipids with altered structure in white matter and a smaller white matter internal capsule. The study did not otherwise evaluate differences in the volume of cerebral structures.

An FTIR study of neonatal rats exposed to a ketogenic diet *in utero* was also performed by Kosiek et al. (28). Consistent with findings from Sussman et al. (24, 26), offspring of ketogenic diet-fed mothers experienced significant reduction in body mass and were found to have neurological developmental delays compared to normal diet offspring. However, switching some of the mothers previously on ketogenic diet to a normal diet at the start of the lactation period allowed the pups to regain most of their weight and neurological function as early as day 14 post-partum. This is consistent with work by Sussman et al. (24) where pups were fostered by dams fed a standard diet, which led to weight recovery at 14 days postnatal. Changes in gross biochemical brain changes in mothers were evaluated using FTIR. Increased lipid and ketone groups containing molecules in the global white matter regions were seen, although this was not statistically different from rats who were fed a normal diet. The findings are interesting considering the fat-dominant composition of breast milk, which implies neonates have an innate propensity for metabolising ketone bodies (29).

The studies so far imply that the use of a ketogenic diet during an otherwise healthy pregnancy could lead to abnormalities in cerebral development, which may lead to behavioural changes, although it is noted that there is no strong evidence to demonstrate long-term behavioural deficiencies following *in utero* exposure to elevated ketone bodies. An avenue that has not been explored is whether ketogenic diets might have an early therapeutic effect in neurodevelopmental conditions starting *in utero.* Indeed, there are clinical case reports to suggest that ketogenic diets in premature neonates with developmental and epileptic encephalopathy can serve to reduce seizure frequency as effectively as anti-seizure medications (30, 31). A potential study might involve using idiopathic models of neuropsychiatric conditions like the EL model, which is used for ASD and epilepsy, or the BTBR mouse model, which is used in ASD studies, and evaluating whether maternal nutritional control could result in improved functional outcomes for offspring. It is acknowledged that clinical studies in prenatal populations would be exceedingly difficult and future work would be focused on animal models.

## Autism spectrum disorder

Autism spectrum disorder (ASD) is a common and complex neurodevelopmental disorder with a wide range of symptoms with varying severity. It is predominantly characterised by social communication difficulties, and repetitive and restricted behaviours. Recognition of these symptoms has significantly improved in recent times, which has led to a sharp increase in diagnoses of ASD with a growing need for therapies. Currently, there are no treatments for ASD as a whole, but some psychological and behavioural therapies exist to improve some of the specific symptoms. Diagnosis and rating of severity is done clinically but the goal standard tool used to support this diagnosis is Autism Diagnostic Observation Schedule, Second Edition (ADOS-2). Other tools such as Childhood Autism Rating Scale, Second Edition (CARS-2) or Autism Treatment Evaluation Checklist (ALTEC) can also be used for assessment of ASD or changes in ASD symptomatology following treatment. ASD is a highly heterogeneous condition with likely multiple pathways involved in its development. However, one of the emerging pathways implicated in ASD pathology is the energy metabolism of microglia, astrocytes and neurons.

Structural abnormalities in the brain development of patients with ASD have been demonstrated in neuroimaging studies, which report trends of slightly decreased brain size at birth followed by rapid volumetric increase in the first years of life that plateaus into older childhood (32–34). Large studies of the Autism Brain Imaging Database Exchange (ABIDE), containing structural MRI of children and adults, conclude that total brain and grey matter volumes are enlarged by 1–2% in ASD patients with specific enlargement of the left anterior superior temporal gyrus, although the statistical significance is uncertain due to the lack of complete matched controls (35).

More recently, mass spectroscopy and magnetic resonance spectroscopy (MRS) studies of metabolites have begun to elucidate variations in neurochemical processes, although results are mixed. In children, reduced levels of N-acetylaspartate (NAA) and gamma-aminobutyric acid (GABA) in all brain regions have been observed studies of ASD patients (36, 37). In some brain-specific studies, drug-naïve adults with ASD do not appear to show any significant differences in metabolite concentrations compared to controls (38). In others, non-medicated, high-functioning adult subjects have been found to have increased levels of choline-containing compounds, creatine, and phosphocreatine in the hippocampal formation, which have also been positively correlated with Buss-Perry Aggression Questionnaire scores (39). Lower tryptophan and altered action of the kynurenine pathway has also been reported in adults (40). Global plasma analysis also shows some variation in ASD metabolites with significantly high levels of arginine and taurine but significantly low levels of 5-oxoproline and lactic acid compared to controls (41).

Bridging the evidence, large scale metabolomic screening studies, often aided by machine learning, have begun to identify a variety of different candidate metabotypes unique to ASD (42); data from 357 children enrolled in the Children’s Autism Metabolome Project (CAMP) yields 34 differentiated metabotypes forming six metabolic clusters, enabling screening with 53% sensitivity and 91% specificity (43). It follows that certain dietary or metabolic interventions might have an impact on regulating ASD symptoms.

A variety of preclinical studies exploring the ketogenic diet for treatment of ASD, dominantly in mice, were found. A range of different murine ASD models are used including pharmacologically induced models (e.g., maternal valproic acid injection), idiopathic models (e.g., BTBR, EL), and genetic models (e.g., Fragile X, Grin1 knockdown), which reflects both the highly heterogenous nature of ASD presentation and the ongoing gaps in a pathophysiological understanding of the disease. All preclinical studies reviewed are characterised by diets that robustly follow a ketogenic protocol (either by high dietary fat or appropriately high MCT supplementation), provide clear confirmation of ketosis via blood or urine testing, and appropriate length diets of at least 4 weeks.

A series of straightforward studies evaluating the effect of ketogenic diets on murine behaviour in ASD models were performed by Ruskin et al. (44, 45) and Castro et al. (46). Two of the studies utilised maternal immune activation via injection of a pharmacological agent, which simulates clinical epidemiological observations of increased incidence of ASD following a maternal bacterial or viral infection during pregnancy (47, 48); Castro et al. (46) injected pregnant mice with 600 mg/kg of valproic acid on day 11 of gestation, while Ruskin et al. (44) injected pregnant mice with 5 mg/kg polyinosinic–polycytidylic acid [poly(I:C)] potassium salt on days 10.5, 12.5, and 14.5 of gestation. Ruskin et al. (45) instead used the EL mouse, a natural model of concomitant autism and epilepsy (49). All studies then fed cohorts ketogenic diets and then subjected mice to similar behavioural tests, as summarised in Table 1. Controls fed non-ketogenic diets were also used as comparators in all studies. Elevated blood BHBs and lowered blood glucose were confirmed in the ketogenic groups of each study. Similar behavioural improvements were also seen in all studies. All studies reported statistically significant reductions in stereotypic behaviour and improvements in sociability for the ASD model fed with a ketogenic diet.

**Table 1:** Summary of preclinical studies.

Author	Theme	Study design	Model (Control)	Intervention	Study groups	Ketosis measured?	Behavioural tests	Other tests	Testing intervals	Results
Kosiek et al. (2022)	PND	Prospective interventional	Pregnant Wistar rats, 12 pups sacrified 2 days postpartum for FTIR, of remaining 7 fed same diet, 6 KD crossover diet	Maternal KD (80% fat)	(1) ND from gestation to PP21 (*n* = 7) (2) KD from gestation to PP2, ND from PP2 to PP21 (*n* = 6) (3) KD from gestation to PP21 (*n* = 5)	Yes, serum	Neurodevelopmental reflex testing (forelimb & hindlimb grasping, righting, hindlimb placing, cliff avoidance, gait test, auditory startle reflex, eye opening)	FTIR microscopy, PCA	PP3-PP21	KD pups experienced significant reduction in body mass with found to have neurological developmental delays compared to ND pups. Switching from KD to ND at start of the lactation period allowed the pups to regain most of their weight and neurological function as early as day 14 post-partum No significant differences in maternal gross biochemical brain changes for KD and ND groups Global delays in reflex testing in KD group
Rugiel et al. (2023)	PND	Prospective interventional	Wistar pups exposed to KD *in utero*	Maternal KD (79% fat, 1% CHO, 8% protein)	(1) Pups exposed to ND (1:6:3 fat:CHO:protein) *in utero* (*n* = unspecified) (2) Pups exposed to KD *in utero* (*n* = unspecified)	Yes, serum	N/A	FTIR microscopy	2, 6 and 14 days post-partum	Biochemical abnormalities not seen in 2 & 6 day old KD exposed rats although relative content of phosphate-containing compounds in white matter higher in cortex of 2 day old KD rats Abnormalities seen in 14 day old rats including increased level of carbonyl groups in cortex and decreased lipids with alterted structure in white matter for KD. White matter internal capusle also smaller in KD group. Differences potentially from disturbance of lipid metabolism from KD exposure
Sussman et al. (2015)	PND	Prospective interventional	CD-1 pups exposed to KD *in utero*, fostered by SD dams	30-day maternal KD 4:1 (67.4% fat, 0.6% carbohydrate, and 15.3% protein wt/wt), SD after birth	(1) Pups exposed to ND (5% fat, 76.1% CHO, 18.9% protein) *in utero*, fostered by ND dam (*n* = 24) (2) Pups exposed to KD *in utero*, fostered by ND dam (*n* = 20)	Yes, serum	Open-field test, forced-swim, exercise wheel	MRI	8–12 weeks	KD diet associated with increased physical activity levels in exercise wheel testing KD mice exhibit less immobile time during forced swim test Increased cerebellar volume, hypothalamic reduction, and corpus callosum reduction relative to total brain volume in KD mice
Sussman et al. (2013)	PND	Prospective interventional	CD-1 embryos exposed to KD *in utero*	30-day maternal KD 4:1 (67.4% fat, 0.6% carbohydrate, and 15.3% protein wt/wt), SD after birth	(1) Embryos exposed to ND (5% fat, 76.1% CHO, 18.9% protein) *in utero* (*n* = 27) (2) Embryos exposed to KD *in utero* (*n* = 32)	Yes, serum	N/A	Imaging (optical projection tomography, MRI)	E13.5, 17.5	Alterations in embryonic organ growth observed KD embryos on average volumetrically larger at E13.5 with larger heart but smaller brain, pharynx, cervical spinal cord, hypothalamus, midbrain, and pons than control KD embryos volumetrically smaller at E17.5 with smaller heart and thymus but larger cervical spine, thalamus, midbrain and pons
Sussman et al. (2013b)	PND	Prospective interventional	CD-1 pups exposed to KD *in utero*, fostered by SD dams	30-day maternal KD 4:1 (67.4% fat, 0.6% CHO, and 15.3% protein wt/wt), SD after birth	(1) Pups exposed to ND (5% fat, 76.1% CHO, 18.9% protein) *in utero*, fostered by ND dam (*n* = 7) (2) Pups exposed to KD *in utero*, fostered by ND dam (*n* = 8) (3) ND biological control (*n* = 15)	Yes, serum	N/A	Imaging (MRI with 3D diffusion tensor imaging), body weight	P11.5, P21.5	Average KD pups exhibit retarded growth by P21.5 compared to SD pup Anatomical differences seen at P11.5 and 21.5 with relative bilateral decrease in cortex, fimbria, hippocampus, corpus callosum and lateral ventricle, but a relative volumetric enlargement of the hypothalamus and medulla
Yan et al. (2024)	ASD	Prospective interventional	Grin1 knockdown mice (WT)	9-week KD 6:1 fat:(proteins + CHO) ratio OR normal chow with BHB supplementation	(1) WT Normal diet (2) WT KD (3) WT Normal diet + BHB (6 mg/mL) (4) Grin1KD Normal diet (5) Grin1KD KD (6) Grin1KD Normal diet + BHB (6 mg/mL)	Yes, serum	Ambulation in novel environment, Y-maze, social motivation	Electron microscopy	12–14 weeks	KD and BHB supplementation reduced hyperactivity in Grin1KD mice. Only BHB improved sensorimotor gating in Grin1KD mice. No difference in social motivation or spatial working memory Grin1KD mice had reduced percentage of myelinated axons in corpus callosum, which was improved with BHB supplementation Mice receiving KD had increased number of abnormal myelinations (e.g., decompaction)
Olivito et al. (2023)	ASD	Prospective interventional	BTBR mice (C57 mice)	5-week KD (PF4390 diet, 67.70% fat, 15.90% protein, 1% CHO)	(1) Control ND (*n* = 8) (2) Control KD (*n* = 8) (3) BTBR ND (*n* = 8) (4) BTBR KD (*n* = 8)	No	Light dark test, 3 chamber test, self-grooming test, novel object recognition test	Cytokine analysis (IL-1β, IL-6 and TNFα), oxidative stress markers, faecal microbiota analysis	5 weeks	KD reduced social-cognitive deficits and repetitive behaviours in BTBR mice Reduced expression of TNFα, IL-1β, and IL-6 in plasma and prefrontal cortex of BTBR mice Lipid peroxidation levels and superoxide dismutase activity altered in BTBR mice brains Relative abundance of putatively beneficial microbiota seen in BTBR and control mice on KD
Uddin et al. (2022)	ASD	Prospective interventional	Shank3+/− zebrafish	BHB or NAD^+^ precursor supplementation in water	(1) Vehicle E3 medium (*n* = unspecified) (2) BHB (1 mM; *n* = unspecified) (3) Nicotinamide nucleotide (50 μM; *n* = unspecified)	No	Zebrabox tracking assay for locomotion	PCR genotyping	2, 3, 5 days post fertilisation	BHB and nicotinamide nucleotide both improve locomotor behaviour Additional HeLa studies indicate β-oxidation of ΒHB promotes mitochondrial elongation by increasing NAD^+^ levels, which then activates SIRT deacetylases acting on key regulators of mitochondrial fusion and fission
Ruskin et al. (2017)	ASD	Prospective interventional	EL mice, idiopathic model of comorbid ASD/epilepsy	3–4 weeks KD [Bio-Serv F3666 (3.0:1) OR Bio-Serv F5140 (6.6:1)]	(1) ND (*n* = 18) (2) 3:1 KD (*n* = 18) (3) 6.6:1 KD (*n* = 18)	Yes, serum	3-chamber test, social transmission of a food preference test, self-grooming	N/A	4 weeks	Sociability was improved and repetitive behaviours were reduced in female mice male behavioural improvements more limited
Ruskin et al. (2017b)	ASD	Prospective interventional	Offspring of C57Bl/6 mice with maternal immune activation via poly(I:C) injection	3–4 weeks KD [BioServ, 6.6:1 fats:(CHO + protein)]	(1) WT ND (*n* = 11) (2) poly(I:C)-treated ND (*n* = 11) (3) poly(I:C)-treated KD (*n* = 11)	Yes, serum	3-chamber test, social transmission of a food preference test, repetitive behaviour	N/A	4 weeks	KD feeding partially or completely reversed all poly(I:C)-induced behavioural abnormalities in males but no effect in females KD reduced blood glucose and elevated blood ketones in both sexes
Castro et al. (2017)	ASD	Prospective interventional	Offspring of Swiss mice with maternal immune activation via VPA injection	49-day KD (70% fat, 24% protein and no CHO)	(1) Saline-treated pups ND (*n* = 8) (2) Saline-treated pups KD (*n* = 8) (3) VPA-treated pups ND (*n* = 8) (4) VPA-treated pups KD (*n* = 8)	No	3-chamber test (sociability index, social novelty preference index), tail-flick, marble-burying (number of marbles buried), self-grooming (time spent)	N/A	49 days	VPA-treated pups fed KD showed improvements in sociability index and social novelty index in 3-chamber test
Carreon-Trujillo et al. (2024)	ADHD	Prospective interventional	Wistar rats with 6-OHDA induced striatal lesions (WT Wistar rats)	Exogenous MCT (10-carbon decanoic acid, i.e., capric acid/C10), 250 mg/kg in DMSO, 26–39 days postnatal	(1) WT saline control (*n* = 8) (2) 6-OHDA saline control (*n* = 8) (3) WT MCT supplement (*n* = 8) (4) 6-OHDA MCT supplement (*n* = 8)	No	Y-maze test (arm entries, % alternation), open-field test (distance moved)	Western blot	25–40 days postnatal	Locomotor hyperactivity (open-field test) and spatial memory (Y-maze) not effected by C10 injection. No change in expression of antioxidant enzymes (GPx1/2, CAT) or Nrf2 expression.
Liu et al. (2023)	ADHD	Prospective interventional	SH rats (WKY rats)	28-day KD (unspecified composition)	(1) WKY ND (*n* = 10) (2) WKY MPH (1.5 mg/kg daily; *n* = 10) (3) WKY KD (*n* = 10) (4) SHR ND (*n* = 10) (5) SHR MPH (1.5 mg/kg daily; *n* = 10) (6) SHR KD (*n* = 10)	No	Open field test (immobility time, total distance moved),	Metabolomic data (ELISA, qRT-PCR, Western blot, 16S rDNA sequencing)	Behavioural testing at 0, 1, 2, 3, 4 weeks; metabolomic testing at 4 weeks	Modest evidence showing improvement in open field test for MPH and KD; MPH shows more significant effect than KD. Significantly increased expression of neurotransmitters (5-HT, NE, AC, cAMP) on KD compared to normal diet, in some cases more than MPH. KD showed alteration in gut microbiota and improved intestinal microbiome balance.
Packer et al. (2016)	ADHD	RCT (double-blinded, placebo-controlled)	Dogs with idiopathic epilepsy (Crossover control)	3-months MCT diet (Normal diet +5.5% MCT, 6.8 kcal/g)	(1) 3-months ND (28% protein, 15% fat, <2% fibre; *n* = 21) (2) 3-months MCT diet (*n* = 21)	Yes, serum Note: MCT diet 0.041 ± 0.004 mmol/L vs. placebo diet 0.031 ± 0.016 mmol/L	C-BARQ	Seizure frequency, body weight	6 months	MCT diet produced reduction in chasing and in stranger-directed fear (p < 0.05) compared with the placebo diet as assessed by C-BARQ. Seizure frequency reduced from 2.67 in placebo to 2.31 seizures/month with MCT diet.

CHO, carbohydrate; DMSO, Dimethylsulfoxide; ELISA, Enzyme-linked immunosorbent assay; KD, ketogenic diet; MCT, medium chain triglycerides; MPH, Methylphenidate; ND, Normal diet; PND, perinatal development; PP, postpartum; PN, postnatal; VPA, valproic acid; qRT-PCR, Real-time quantitative PCR RCT: randomised controlled trial; SH, spontaneously hypertensive; WKY, Wistar Kyoto; WT, wild type.

Some sex differences were noted in the studies by Ruskin et al.; Castro et al. (46) only performed testing on male pups. In the EL model, female mice were found to require a less strict ketogenic diet (3:1 vs. 6.6:1 fat:carbohydrate) to yield the same improvements in sociability and repetitive behaviour, while in males improvements in sociability and repetitive behaviour were less pronounced with the ketogenic diet treatment (45). Similarly, in the poly(I:C)-induced ASD model, males fed a control diet had higher asocial and repetitive behaviours than females, which were ameliorated to a greater extent with a ketogenic diet than females (44). Sex differences in ASD are an under-researched, and to some extent contentious, aspect of the condition. It is uncertain to what degree the male preponderance in ASD, as reflected by a male:female diagnostic ratio of 4.5:1 (50), should be attributed to sex-specific genes, hormonally mediated sex-specific pathways, or even social biasing in diagnostic tools (51–53). The preclinical studies here highlight some nuanced sex differentiation of various ASD models, severity of symptoms, and importantly response to treatment.

On top of behavioural testing, some other preclinical studies performed further testing to elucidate some of the underlying mechanistic processes of ketogenic therapies. Yan et al. (54) compared a ketogenic diet to a normal diet with BHB supplementation in attenuating behavioural symptoms of Grin1 knockdown (Grin1KD) mice, performing electron microscopy of the corpus callosum to visualise changes in axon morphology. The Grin1KD model is characterised by a 90% reduction in Grin1 mRNA, GluN1 protein, and NMDAR function, which in turn elicits cognitive deficits, reduced social motivation, impaired sensorimotor gating and seizures. Both the ketogenic diet and BHB supplementation raised serum BHB levels, although the exogenous supplementation was better tolerated. In contrast to other preclinical testing, neither ketogenic treatment yielded significant improvements in behavioural testing for social motivation, working memory, or short-term spatial memory, but BHB supplementation did modestly improve the prepulse inhibition of acoustic startle response (PPI) in sensorimotor gating tests. Electron microscopy showed that BHB supplementation modestly increased the percentage of myelinated axons in Grin1KD mice. Unexpected increases in the percentage of myelin decompaction abnormalities were seen in the ketogenic diet group. As decompaction is generally associated with neurodegenerative disease, this observation is at odds with the generally held understanding that the ketogenic diet imparts a neuroprotective effect by promoting myelination (55–57). Discrepancies observed in behavioural testing may be partially attributed to the selection of the Grin1KD model, which is dominantly characterised by NMDA receptor dysfunction and is less well-established for ASD despite exhibiting some behavioural similarities; rather, reduced NMDA receptor function has more strongly been implicated in schizophrenia (58, 59).

Olivito et al. (60) characterised the microbiota and cytokine landscape of BTBR mice, an idiopathic model of ASD, fed a ketogenic diet for 5 weeks 22 days after weaning. Similar to studies by Ruskin et al. (44, 45) and Castro et al. (46), administration of the ketogenic diet was associated with significant reductions in social deficits, repetitive behaviours, and cognitive impairment. Following behavioural testing, mice were sacrificed, and brain samples were evaluated for IL-1β, IL-6 and TNFα as well as oxidative stress markers. The ketogenic diet was associated with reduced expression of IL-1β, IL-6 and TNF*α* in plasma, the prefrontal cortex, and hippocampus. Reduced oxidative stress was evidenced by reduced thiobarbituric acid reactive (THBAR) substances in the prefrontal cortex and hippocampus after the ketogenic diet. The intervention also appeared to increase α-diversity in faecal microbiota of the ASD model when compared to the ASD model fed on a control diet. At a Phylum level, some alterations in the relative abundance of certain speciation was seen following the ketogenic diet, although the functional implications of this are not understood.

An association mitochondrial dysfunction and ASD has been postulated since the 1980s after observations that patients with autism frequently had elevated lactate levels (61), which has in turn prompted a variety of associations between structural and functional abnormalities and ASD (62, 63). Uddin et al. (64) evaluated exogenous BHB effects on mitochondria and autism-like behaviour in shank3+/− zebrafish model of ASD. They found that BHB administration promoted mitochondrial elongation via increase in NAD+ levels. Mitochondrial elongation is associated with improved mitochondrial function and their longevity (65). BHB additionally improved locomotor behaviour in the shank3+/− zebrafish. Not only does this support the mitochondrial dysfunction in autism hypothesis but it also points towards a potential molecular mechanism by which ketone bodies improve ASD symptoms.

Three studies stem from a non-randomised, open label clinical trial (NCT02477904) assessing the effectiveness of the ketogenic diet for treating ASD. Children aged 2–21 with a confirmed diagnosis of ASD were placed on an MCT-supplemented gluten-free ketogenic diet, where 20% of caloric intake was derived from MCTs, carbohydrates were limited to 25 g/day, and the remainder of energy were derived from fats and protein. A total of 46 patients were initially recruited but only 23 started the diet intervention with further dropouts at the various follow-up periods. Separate studies were published addressing different outcomes and as such the follow-up periods and total number of participants included was slightly different.

The first study evaluated behavioural outcomes using ADOS-2 and CARS-2 at 3 (n = 15) and 6 (n = 10) months after initiation of the diet (66). ADHD was comorbid in 66.67% of patients and 73.4% were on existing medications including psychostimulants (e.g., methylphenidate) and anti-epileptics (e.g., diazepam, lamotrigine). Baseline ADOS-2 scores for all participants were either moderate or high. Compliance with the diet was confirmed with serum BHB levels, which were elevated from baseline (0.196 mmol/L, *p* = 0.025) to 1.351 mmol/L at 3 months. Total and social affect ADOS-2 scores were significantly improved at 3 and 6 months but restricted repetitive behaviour scores were not significantly improved. CARS-2 scores were also significantly improved after 3 months.

The results observed by Lee et al. (66) complemented two other clinical trials that evaluated changes in ASD behaviour in children following administration of a ketogenic diet. Evangeliou et al. (67) used CARS to evaluate changes in autistic behaviour of children aged 4–10 years fed a ketogenic diet (30% MCT, 30% cream, 11% saturated fat, 19% carbohydrates, 10% protein) for 6 months. Ketosis was confirmed with serum BHBs. Of the 18 patients, 2 patients saw a significant reduction in their CARS score (>12 units), 8 patients saw an average improvement (8–12 units), and 8 patients saw a minor improvement (2–8 units). In a case–control study, El-Rashidy et al. (68) trialled two diets including a modified Atkins diet comprised of 60% fat, 30% protein, and 10% carbohydrate and a non-ketogenic gluten-free casein-free (GFCF) diet. The average CARS score in the ketogenic Atkins diet group reduced by 8 points from 41.70 ± 5.52 pre-diet to 33.70 ± 4.2 post-diet (*p* = 0.0001), similar to Evangeliou et al.’s (67) study.

A second study evaluated metabolites from bloods of the 3-month follow-up cohort (n = 17) using gas chromatography–mass spectroscopy (GC–MS), ^1^H NMR spectroscopy, and inductively coupled plasma–mass spectrometry (ICP-MS) (69). Metabolite results were compared against bloods from typically developed control children (n = 10). It was found that an ASD metabolic profile based around attenuations of glutamate, galactonate, and glycerol could be discriminated with 88% accuracy, with baseline ASD featuring higher concentrations of galactose intermediates, gut microbe-derived trimethylamine N-oxide and N-acetylserotonin, and lower concentrations of 3-hydroxybutyrate and selenium. Ketogenic diet treatment led to increases in ketone bodies and selenium. Correlating with ADOS-2 scores, it was found that higher behavioural responders also had higher concentrations of BHB and ornithine and lower galactose following the ketogenic diet. This provides early evidence for potential biomarkers and a metabolic model for various degrees of ASD severity.

Alterations to the gut biome and cytokine expression in the same cohort of ASD were reported in a later study (70). In this report, a reduced number of children submitted non-fasted blood (*n* = 11) and stool (*n* = 7) samples before and after 4 months on the ketogenic diet. RNA and DNA were extracted from stool samples and sequenced while a series of cytokines were tested from blood. Diet compliance was also confirmed via plasma AcAc and BHB levels. Unlike the study by Mu et al. (69), no data from neurotypical controls was studied. 4-months of ketogenic diet showed changes in microbial diversity at lower taxonomic levels but not at phylum-level abundance, suggesting some improvement in the distribution of microbes. A trend towards decreasing expression of inflammatory cytokines were seen with significant decreases in IL-1β and IL-12p70. BDNF expression was also reduced alongside expression of selected associated miRNAs linked to regulating cerebral BDNF activity.

The remaining clinical studies identified are various case reports. Spilioti et al. (71) published a broad series of case studies encompassing 187 patients with ASD. Cohort members were subjected to glucose challenge testing. 16 candidates with pathologically elevated serum BHB and lactate were considered as candidates for mitochondrial disease, although this wasn’t confirmed via muscle biopsy. Of these, 6 were initiated on a ketogenic diet of unspecified composition and duration. All of the patients selected had comorbid symptoms of ADHD. At a 6-month follow-up, all patients exhibited a reduction in CARS score, with one patient experiencing complete remediation of symptoms. Another case study focusing on a 6-year old with autism and epileptic discharges was placed on a ketogenic diet based on 18FDG PET revealing glucose hypometabolism in the brain (72). A month after initiation of treatment, behaviour and intellect appeared to improve, particularly for hyperactivity and attention span. Follow-up 18FDG PET showed changes in 18F-FDG uptake across the whole cerebral cortex. This case study illustrates early diagnostic potential for PET in autism with visible changes following administration of a ketogenic diet. Other studies attempting to evaluate the diagnostic utility of PET in ASD exist in a research capacity, but results remain mixed (73, 74).

Herbert & Buckley (75) also reported a case study using an MCT ketogenic diet on a child with ASD and seizures. Over the period of some years, a reduction in CARS was observed such that the patient was considered nonautistic at the final follow-up. EEG also revealed significant attenuation of seizure activity. While the almost complete amelioration of symptoms in this case is compelling, such a dramatic result is not broadly supported by any of the clinical trials reviewed thus far.

Similar to preclinical studies on the ketogenic diet and ASD, the clinical studies reviewed are characterised by diets that follow an unambiguous ketogenic protocol, provide clear confirmation of ketosis via blood or urine testing, and appropriate length diets of at least 4 weeks. Some common limitations include the small cohort sizes, lack of blinding, and lack of studies trialling alternative exogenous ketone preparation like ketone esters. Clinical studies indicate while there are some dropouts the diets are usually well tolerated. No studies using ketone salts or other exogenous preparations were reported, which could be an area of future.

Perhaps most compelling from the clinical work are the unusually large improvements seen in CARS and ADOS. Both CARS and ADOS are primarily diagnostic tools utilised during early consultations to positively diagnose ASD. The tools are not routinely used during management to evaluate changes to or improvement in autistic symptoms, as they have been used in the clinical studies reviewed here. As such, the degree to which clinician might typically expect to see changes in CARS or ADOS scores in uncertain. However, it is generally considered that ADOS scores do not fluctuate spontaneously and are typically stable. In short periods of time (i.e., 4-months for children aged 3–13 years old, 8 months for adolescents >15 years old), test–retest reliability studies do not show substantial changes in ADOS-2 scoring (76). Over periods of 3-years, one study shows that ASD patients may see statistically significant changes in ADOS-2 scores, especially in the social domain, although it might be criticised that over time improvements in social functioning could partially be attributed to learned behaviour (77). However, when discriminating for IQ, patients with IQ < 70 rather tended to exhibit decreases in ADOS scores over time (78). It might generally be stated that clinically speaking, there is not typically an expectation for ADOS or CARS scores to reduce, even with supportive therapy across a patient’s lifetime. For the duration of the ketogenic diets employed here (i.e., <6 months), the dramatic improvements in ADOS and CARS scores are particularly remarkable and not even reliably replicated by first-line treatment recommendations in countries with healthcare systems employing evidence-based reviews of treatment. While important to consider alternative explanations such as social training or the placebo effect of non-blinded studies, related studies reporting improvements in ADOS or CARS scores also employing dietary changes such as probiotic treatment (79), omega-3 polyunsaturated fatty acids (80), or vitamin D imply that treatments addressing metabolic and nutritional changes may have a genuinely beneficial effect for patients with ASD. A potential weakness of the studies reviewed may be that ADOS and CARS are primarily diagnostic tools and do not necessarily evaluate severity well in the short-term; tools like the Global Assessment of Functioning (GAF) scale or Clinical Global Impression (CGI) scale might be better suited to this end. However, the results of the studies reviewed provide compelling evidence for the therapeutic use of ketogenic diets and supplementation in ASD.

## ADHD

Attention deficit and hyperactivity disorder (ADHD) is the commonest childhood neurodevelopmental disorder affecting between 3–9% of school aged children (81). ADHD is characterised by a variety of symptoms across three main domains: inattention, hyperactivity and impulsivity. It is a highly heterogeneous condition where patients may have differing burden of symptoms from each domain. The majority of those diagnosed in childhood will have symptoms that persist into adulthood meaning the burden of disease is typically lifelong. The consequences of untreated ADHD are broad and can also be severe; ADHD is more likely to be comorbid with other mental diseases (82) including anxiety (83), depression (84), substance misuse disorders (85), and psychosis (86). Concurrently, risk of poorer educational outcomes, criminality and suicide are all significantly increased if ADHD is not treated (87–89). Currently the main mode of treatment is pharmacotherapy using stimulant or non-stimulant medication affecting noradrenergic or dopaminergic systems in the central nervous system. Unfortunately, these medications come with a wide range of side effects (90), meaning there is a strong demand for alternative treatment options with more benign adverse effects.

The neuroenergetic hypothesis of ADHD proposed by Russell et al. (91) posits that astrocyte functional deficiencies due to impaired energetic supply are a potential cause of inefficient and inconsistent neuronal performance, either via deficient ATP production leading to slowed restoration of ionic gradients across neuronal membranes and delayed neuronal firing, or insufficient lactate supply in oligodendrocytes causing impaired fatty acid synthesis and myelination of axons during development. An early evidence-base for a neuroenergetic model of ADHD has been established in recent preclinical studies showing that certain rat models of ADHD exhibit distinct modifications of metabolic pathways for energy metabolism and oxidative stress when compared to controls (92). In humans, urinary metabolite studies also show distinct metabolic profiles in children with ADHD compared to healthy controls, in particular tyrosine, leucine, amino acid, and fatty acid metabolic pathways (93). Notably, urine concentration of the branched chain fatty acid 3,4-methylenepimelic acid was decreased while the fatty acid derivatives aminocaproic acid and 3-methylazelaic acid were increased in ADHD patients (93). Blood studies have shown that ADHD scores in adults are positively correlated with higher concentrations of stearic and monounsaturated fatty acids (94, 95). In light of existing evidence showing alterations to fatty acid metabolic pathways, it is possible that ketosis, as induced by dietary or exogenous interventions, could potentially offer a regulating effect on ADHD symptoms.

A handful of papers evaluating the use of a ketogenic diet in models of ADHD have been compiled. At present, there are no interventional clinical studies that directly evaluate the impact of a ketogenic diet or exogenous ketone supplementation on ADHD symptoms. A study by El-Baaki et al. (96) found that 5-weeks of diet modification could improve ADHD symptoms as measured by Conner’s Parent Rating (CPR) scale, but the diet was not ketogenic and featured a composition of 45–65% carbohydrates, 10–35% protein, and 20–35% fat. The closest clinical evidence can be found in assorted case reports trialling ketogenic diets where attenuation of ADHD symptoms is mainly mentioned as a secondary outcome. Many of these case studies rather focus on patients with epilepsy, a condition for which the ketogenic diet is well-established as a treatment option and that has a reasonably high prevalence of comorbidity with ADHD (97). For example, a case series by Ballara Petitbo et al. (98) describing 17 patients with epilepsy featuring eyelid myoclonia found that 2 patients treated with a ketogenic diet of unspecified composition and length experienced improvement of seizures and comorbid neuropsychiatric symptoms, where around 60% of neuropsychiatric symptoms in the cohort were attributed to ADHD diagnoses.

While not specifically a ketogenic diet, a recent population study of nutrient supply data from over 150 countries from 1990 to 2018 noted an interesting trend whereby higher fat intake was associated with decreased ADHD disease burden while carbohydrate and protein supplies conferred minimal influence (99). Further, an increased fat:carbohydrate ratio was associated with lower incidence of ADHD, as was an increase in plant-based fat supply. The data appears to loosely provides some evidence that a move towards high-fat ketogenic-esque diets, where there is a higher likelihood of achieving ketosis, is associated with decreased ADHD burden. Plant-based fats are higher in mono- and polyunsaturated fatty acids. It has previously been reported that 4:1 ketogenic diets utilising polyunsaturated fats, as opposed to saturated fats that comprise animal-based fats, induce more pronounced ketosis in the same period of time (serum BHB: 8.4 mg/dL polyunsaturated fat diet vs. 3.1 mg/dL saturated fat diet, *p* < 0.05) (100), which may provide some explanation for the associations observed by Ni et al. (99).

A small number of preclinical studies have directly evaluated the therapeutic potential of the ketogenic diet in ADHD. However, it might be considered that none of these provide strong evidence for the claims being made due to oversights in experimental design. Packer et al. (101) conducted the only randomised controlled trial claiming to demonstrate reduction of ADHD-like behaviours in dogs. Dogs with idiopathic epilepsy, an ADHD model also used in rats based on comorbid symptoms including hyperactivity and impulsivity, were fed a ketogenic diet of kibble supplemented with MCT oil. Compared to the crossover control group, the authors claimed that the MCT diet reduced some ADHD-like behaviours as assessed by the Canine Behavioural Assessment and Research Questionnaire (C-BARQ).

Although previously included in a review by Kraeuter et al. (102) focusing on the therapeutic potential of ketosis as evidence that ketogenic treatments might be beneficial in reducing hyperactive behaviours, it is important to consider to what extent canine models are representative of humans. Canines derive their energetic requirements dominantly from protein and have no essential nutritional need for carbohydrates (103). As such, the relative proportions of a ketogenic diet should look substantially different for humans and dogs; indeed, the ketogenic diet used by Packer et al. (101) only consisted of 5.5% MCTs accounting for only 10% of the total caloric intake, which is considerably less than typical MCT diet formulations in humans where MCTs generally comprise 50–60% of caloric intake.

Further, there is still uncertainty surrounding how physiological ketosis might be defined in canines. Packer et al. (101), as well as a separately published study utilising the same dataset (104), purport that a statistically significant increase in serum BHB is observed in the group receiving the ketogenic diet. However, in relative terms the serum BHB of the test group (0.041 ± 0.004 mmoL/L) is only 32% greater than the placebo diet (0.031 ± 0.016 mmoL/L, *p* = 0.028) compared to the 100–500% increase expected for physiological ketosis in humans (based on normal levels = 0.5 mmoL/L and physiological ketosis = 1–3 mmoL/L). Given that studies of canines with diabetic ketosis and ketoacidosis exhibit average serum BHB levels of upwards of 5 mmoL/L (105, 106), it is possible that the subjects are not in a ketotic state and therefore any behavioural improvements cannot be attributed to the effects of a ketogenic diet.

Two recent studies by Carreón-Trujillo et al. (107) and Liu et al. (108) evaluated the effect of ketogenic therapies in Wistar rats. A fundamental limitation of both these studies is that physiological ketosis was not confirmed via serum BHB levels or otherwise. Additionally, the study by Liu et al. (108) did not specify the composition of the ketogenic diet, which limits comparison of the intervention.

Carreón-Trujillo et al. (107) used Wistar rats lesioned unilaterally with 6-hydroxydopamine (6-OHDA) injection as an ADHD model. Striatal 6-OHDA-lesioning is a well-established murine ADHD model characterised by significant dopaminergic denervation of the striatum and subsequent reactive serotoninergic hyperinnervation (109). The model offers clear hyperlocomotor and attention deficits that respond to methylphenidate and amphetamine (110), making it an effective model for researching treatments. Unlike other studies using MCTs where the ketogenic supplements are fed to the subjects, this study used daily subcutaneous injection of decanoic acid (250 mg/kg) for a period of 14 days. As serum KB levels were not measured, it is not known whether the injections produced a state of physiological ketosis. In Y-maze and open-field testing, no statistically significant difference in locomotor activity or spatial memory was observed between the four study groups (see Table 1). No significant difference was seen in the expression of antioxidant enzymes (including catalase and glutathione peroxidase-1/2) in the striatum, prefrontal cortex, hippocampus or cerebellum or the phase II transcription factor Nrf2 in the prefrontal cortex and cerebellum. It is noted that dopaminergic levels in the striatum were not measured. Dosages for subcutaneous injection of MCTs are not well understood as few studies validate serum or plasma BHB levels following administration. Some studies reporting positive anti-convulsant effects of capric acid injections in mice use concentrations some 30 times higher (i.e., 1.7–8.6 g/kg) than the concentrations used in this study (111). It is possible that dosing for subcutaneous injection might have to be substantially higher than oral administration as transport to the liver via cutaneous tissue is likely to be slower than via the digestive tract.

Liu et al. (108) used spontaneously hypertensive rats (SHR) as an ADHD model to evaluate the effects of a 28-day ketogenic diet of unspecified composition. The SHR model has been shown to have a distinguishable brain, blood, and urine metabolome compared to controls (92). The ketogenic diet was also compared against a group treated with methylphenidate. Unlike Carreón-Trujillo et al. (107), Liu et al. (108) reported improved performance in open-field testing, albeit not as pronounced as when treated with methylphenidate. A strength of the study is its interrogation of dopaminergic system gene expression, as ADHD is partially characterised by hypofunction of the dopaminergic system. They found that the biogenic amines 5-HT and NE, downstream G protein effectors and second messengers AC and cAMP, and DA transports and receptors (DAT, DRD1, DARPP32) were lower at baseline in the SHR rats and significantly higher in both the ketogenic diet and methylphenidate-treated groups indicating that the ketogenic diet can attenuate expression of neurotransmitters at a protein level. In the case of AC, NE, DRD1, DAT, PKA, and DARPP32, expression in SHR rats was higher when treated with the ketogenic diet than with methylphenidate. They also noted significant changes in the composition of gut microbiota with and without ketogenic diet treatment, which is consistent with other studies also reporting variability in the microbiota of patients with ADHD (112), although the manner in which this influences behavioural symptoms is not yet understood (Table 1).

Compared to studies for ASD, evidence supporting the use of the ketogenic diet in ADHD is less compelling. In particular, the lack of rigorous biochemical data characterising ketosis (incl. Serum levels of ketone bodies, serum glucose, lipid profiles) and variability in dietetic protocol limits the clinical applicability of studies. Considering the significant methodological limitations of the studies reviewed, it is possible that the therapeutic effects of ketosis in ADHD are simply yet to be elucidated; the etiological overlap of ASD and ADHD, coupled with the moderately strong evidence that ketogenic diets have remarkable therapeutic value in ASD patients imply that ADHD patients are also likely to benefit to some extent from ketogenic protocol.

## Discussion

The following review has explored literature describing the therapeutic potential of the ketogenic diet or exogenous ketone supplementation in the treatment of neurodevelopmental disorders, namely abnormalities proliferating during the perinatal period, ADHD, and ASD. Results from preclinical perinatal studies provide some early results showing that feeding mothers a ketogenic diet tends to deleteriously impact the birth weight (24, 26–28), which might be considered a global proxy for growth retardation and risk of neurodevelopmental delay (113), relative brain volume (25, 26), and performance in behavioural testing of offspring (25). Notably, as the studies all utilise healthy or neurotypical murine models, there is little evidence indicating whether maternal ketosis might have a therapeutic effect in populations at risk of neurodevelopmental deficits. This would be a topical area of future research as the current view on perinatal ketogenic diets is largely negative.

The most common study design in both preclinical and clinical studies involves implementing a ketogenic protocol (either dietary or supplemented) in a population and observing behavioural changes via either behavioural testing in preclinical work or a standardised tool in clinical work (Table 2). In ASD, there is reasonable evidence from a series of straightforward studies associating ketosis, as achieved by either the ketogenic diet or MCT supplementation, with behavioural improvement (44–46, 66, 67). In ADHD, associations between ketosis and behavioural improvement were less clear, but it is also noted that the studies reviewed have a number of issues in experimental design that perhaps limit the scope of conclusions.

**Table 2:** Summary of clinical studies.

Author	Theme	Study design	Population	Intervention	Comparator	Ketosis measured?	Outcome (Behavioural)	Outcome (Other)	Follow-up period	Results
Allan et al. (2024)	ASD	Clinical Trial, Prospective interventional pilot	Children with ASD (*n* = 11 blood samples, *n* = 7 stool samples)	KD (gluten-free with MCT, CHO max 20-25 g/day, 20% MCT oil)	No control group	Yes, plasma	N/A	RNA/DNA from stool, IL-1b, IL-4, IL-5, IL-6, IL-8, IL-10, IL-12p70, IL-17A IFN-γ, BDNF, TNF-α, PDGF, MIP-1β, and VEGF-A from blood, KB assay	4 months	KD decreased IL-12p70, IL-1b and BDNF. Changes in the gut microbiome, increased expression of butyrate kinase in the gut, and altered levels of BDNF-associated miRNAs in the plasma.
Mu et al. (2020)	ASD	Clinical Trial, Prospective interventional pilot	Children with ASD (*n* = 17)	3-month KD (gluten-free with MCT, CHO max 20-25 g/day, 20% MCT oil)	Non-ASD control, aged 2–21 (*n* = 10)	Yes, plasma	ADOS-2, CARS-2	Blood GC–MS, 1H NMR,	3 months	Glutamate, galactonate, and glycerol discriminated ASD with 88% accuracy at baseline Ornithine concentration negatively correlated with the ADOS-2 overall score and the social affect score AcAc negatively correlated with the comparison score, ADOS-2 and the social affect score KD restored lower selenium levels in ASD. correlation analysis identified a novel negative correlation between the changes in selenium and behaviour scores. High responders had greater concentrations of BHB and ornithine, with lower galactose levels
Lee et al. (2018)	ASD	Clinical trial, pre-post, open-label, observer-blinded	Children with ASD (*n* = 15 for 3 months, *n* = 10 for 6 months)	Modified KD/GF/MCT protocol, limited total net CHO daily intake to 20–25 g, 20% MCT	Pre-intervention	Yes, serum	ADOS-2, CARS-2	N/A	1, 3, 6 months	Improved core autism features on ADOS-2 after 3 months No difference in restricted and repetitive behaviour score after 3 months Substantial improvement (>30% decrease total score) observed in 6, moderate improvement (>3 units) in 2 participants, and minor/no improvement in 7 Sustained improvement in total ADOS-2 and social affect sub-domain scores in 10 patients at 6 months but no significant improvement in restricted and repetitive behaviour scores Significant improvements in CARS-2 items after 3 months of KD in imitation, body use, and fear or nervousness
El-Rashidy et al. (2017)	ASD	Case–control study	Children aged 3–8 with ASD (*n* = 45)	(1) Modified Atkins diet (60% fat, 30% protein, 10% CHO, max CHO 8–10 g/day; *n* = 15) (2) GFCF diet (*n* = 15)	Normal diet (*n* = 15)	Yes, urine	CARS, ATEC	N/A	6 months	Modified Atkins and GFCF groups showed improvement in ATEC & CARS Modified Atkins scored better in cognition & sociability compared to GFCF
Herbert & Buckley (2013)	ASD	Interventional case study	Child with ASD (*n* = 1)	GFCF diet (1.5:1) with MCT supplementation (unspecified concentration)	N/A	No	CARS	EEG	14 months	CARS score decreased from 49 to 17, representing a change from severe autism to nonautistic IQ increased 70 points Initial EEG after seizure onset showed lengthy 3 Hz spike–wave activity; 14 months after diet initiation, patient was seizure free, EEG showed only occasional 1–1.5 s spike–wave activity without clinical accompaniments.
Spiloti et al. (2013)	ASD	Interventional case studies	Children with ASD (*n* = 6)	KD (unspecified)	N/A	Y serum	CARS	N/A	6 months	Reduction in CARS scores for all patients
Zarnowska et al. (2011)	ASD	Interventional case study	Child with ASD (*n* = 1) and significant glucose hypometabolism in the brain	Low glycaemic index treatments (LGIT; 40–60 g per day (approx. 10% of daily calories) and restricted carbohydrates from foods with known high GI scores)	N/A	Yes, serum & urine	CARS	18FDG PET	11, 16 months	Behaviour and intellect improved (in regard to hyperactivity, attention span, abnormal reactions to visual and auditory stimuli, usage of objects, adaptability to changes, communication skills, fear, anxiety, and emotional reactions) 18FDG PET, at 12 months, revealed decreased uptake markedly and diffusely in the whole cerebral cortex with a relatively low reduction in basal ganglia in comparison to the pre-KD assessment.
Evangeliou et al. (2003)	ASD	Clinical trial	Children with ASD (*n* = 23)	6-month KD, John Radcliffe diet (30% MCT oil, 30% fresh cream, 11% saturated fat, 19% CHO, 10% protein) 4-weeks on, 2-weeks off	No control group	Yes, urine and serum	CARS	N/A	6 months	60% of participants saw improvements in CARS Significant improvement (> 12 units) recorded in 2 patients (pre-Scale: 35.00 ± 1.41 [mean ± SD]) Average improvement (> 8–12 units) in 8 patients (pre-Scale: 41.88 ± 3.14 [mean ± SD]) Minor improvement (2–8 units) in 8 patients (pre-Scale: 45.25 ± 2.76 [mean ± SD]).
Ni et al. (2024)	ADHD	Retrospective observational	Global data (150 countries, 1990–2018)	Plant-based fat supply	Low plant-based fat supply	No	N/A	ADHD prevalence and incidence	1990–2018	Modelling suggested the interactive effects of nutrients and socioeconomic status on ADHD Fat, especially plant-based fat supply, is associated with decreased ADHD disease burden Associations were conserved across sexes and ages and were not confounded by the total energy supply or GDP/capita
Petitbo et al. (2023)	ADHD	Retrospective observational case series	Epilepsy with eyelid myoclonia patients and co-morbid ADHD aged 1–10 years (*n* = 2)	KD, unspecified composition	Pre-intervention	No	N/A	Eyelid myoclonia control	Unspecified	Eyelid myoclonia controlled on KD

ADOS, Autism Diagnostic Observation Schedule; ATEC, Autism Treatment Evaluation Test; CARS, Childhood Autism Rating Scale; EEG, Electroencephalogram; GFCF, Gluten-free casein-free; GI, Glycaemic index; LFT, Liver function test.

Some studies also collected data relating to metabolites, biomarkers, DNA expression, and gut microbiota. In ASD, reductions in cytokines were observed (60, 70). In both ASD and ADHD, some evidence of attenuation in DNA expression were observed (64, 108) as well as changes in gut microbiota composition (60, 108). The addition of neuroimaging provided some insight into the spatial distribution of certain biomarkers (60), changes in cerebral metabolism (71), and changes in volume of different cerebral areas. While these studies show some interesting associations, it remains largely unclear what the potential therapeutic implications are. This is especially the case in work where the changes seen aren’t necessarily positive or negative, for example, changes in microbiota speciation.

An overarching challenge of the current body of literature is the high degree of heterogeneity in study design, which limits meaningful comparison of results. This includes variability in the intervention used (i.e., ketogenic diet, ketone body supplement), the degree of ketosis observed, and the outcomes measured (e.g., behavioural testing, biomarkers). There is subsequently ample opportunity for researchers to pursue clinical studies as there remain a lot of low hanging fruit in the area, especially considering how clinically safe and tolerable ketogenic regimens are in patients of all ages.

To an extent, the generalisability of the pathophysiology of neurodevelopmental conditions in animal models to humans limits the utility of insight gleaned from preclinical work. While the phenotypic deficits of certain models may appear to mimic the effects of a given condition, the mechanistic processes may be different. Lesion-based models that ablate particular cerebral regions or cells may overestimate the extent of deficits such that no compensating mechanisms are available; it is interesting to note, for example, that both the NMDA hypofunction ASD model, which utilises an extreme 95% reduction of the NMDA receptor 1 subunit (114), used by Yan et al. (54) and the 6-OHDA lesion of dopaminergic receptors used by Carreon-Trujillo et al. (107) report being the most refractory to the ketogenic intervention. The discrepancy between preclinical versus clinical interpretations of neurodevelopmental disease should also be addressed. It is common for preclinical models to focus on symptomatic behaviour across a limited number of domains. In practice, few if any models simultaneously provide face, construct, and predictive validity of neurodevelopmental disorders; for example, while many ADHD models exhibit hyperactivity, few also exhibit inattention and impulsivity. In clinical practice, ADHD is diagnosed where several symptoms across the triad of inattention-hyperactivity-impulsivity are present persistently, while ASD is characterised by deficits in social interaction and communication across multiple contexts; in both cases preclinical studies fail to capture the broadband nature and persistence of symptoms. Researchers should thus be mindful of discussing the limitations of models used.

Although the data supporting the use of a ketogenic intervention for neurodevelopmental disorders is mixed, the studies remain in their infancy. The safety and tolerability of ketogenic diets and ketogenic supplements is such that there would be value in exploring further clinical studies. Particular priorities including establishing their therapeutic benefit in ADHD and high risk perinatal populations as well as studies that directly compare the behavioural benefits of a ketogenic diet versus exogenous ketone supplementation.
